# Δ-β2M as a dynamic biomarker for predicting ICU admission after CAR T-cell therapy

**DOI:** 10.1007/s00277-026-07075-0

**Published:** 2026-05-26

**Authors:** Alexander C. Angleitner, Markus Maulhardt, Kristian Thomson, Judith Büntzel, Gerald Wulf

**Affiliations:** Department of Hematology and Medical Oncology, University Medical Center Göttingen, Robert-Koch-Strasse 40, Göttingen, Lower Saxony 37075 Germany

**Keywords:** CAR T, CRS, ICANS, Risk prediction, Risk factors, Hematological neoplasia

## Abstract

CAR T-cell therapy is associated with life-threatening toxicities requiring intensive care unit (ICU) admission. Reliable biomarkers for early risk stratification are lacking. We evaluated Δ-β2-microglobulin (Δ-β2M) as a dynamic biomarker for predicting ICU admission after CAR T-cell therapy. Single-center retrospective study with a derivation cohort (2020–2023, *n* = 97) and two temporally independent validation cohorts (2024, *n* = 38; 2025, *n* = 23). Δ-β2M was calculated as the change between infusion (T2) and day +3 (T3). Primary endpoint was ICU admission. Predictive performance was assessed using receiver operating characteristic (ROC) analysis, calibration (Hosmer-Lemeshow test, Brier score), and decision curve analysis. Complete Δ-β2M data were available in 54 (derivation), 17 (2024), and 17 (2025) patients. Δ-β2M demonstrated consistent discrimination for ICU admission across all cohorts (derivation AUC 0.67; 2024 AUC 0.70; 2025 AUC 0.82), but not for immune effector cell associated neurotoxicity syndrome (ICANS) (AUC 0.53). The model showed good calibration (Hosmer-Lemeshow *p* = 0.073; Brier score 0.232), confirmed by cross-validation (Brier score 0.236). Decision curve analysis revealed positive net benefit across clinically relevant thresholds (0.20–0.70). Baseline parameters (albumin, CRP, NT-proBNP) lacked consistent validation. Δ-β2M is an easily measurable dynamic biomarker associated with ICU admission after CAR T-cell therapy, with good calibration and clinical utility. These findings support further evaluation of early biomarker kinetics in toxicity risk prediction models.

CAR T-cell therapy has transformed outcomes in several hematologic malignancies [1–3], but remains limited by potentially life-threatening toxicities such as cytokine release syndrome (CRS) and immune effector cell associated neurotoxicity syndrome (ICANS) [4, 5]. Despite multiple proposed clinical and laboratory predictors, no biomarker has proven reliable enough for routine risk stratification [6]. Dynamic biomarkers have improved risk assessment in other inflammatory and oncologic settings, suggesting that early biomarker kinetics may enhance toxicity prediction following CAR T-cell therapy. We therefore evaluated baseline and dynamic laboratory parameters associated with intensive care unit (ICU) admission after CAR T-cell therapy and examined their reproducibility across temporally independent validation cohorts. Furthermore, we assessed the calibration and clinical utility of Δ-β2M using decision curve analysis.

We conducted a single-center retrospective study at the University Medical Center Göttingen, approved by the local ethics committee (approval number 26/3/25). The derivation cohort included 97 adult patients undergoing CAR T-cell therapy between January 2020 and December 2023. Temporal validation was performed in independent cohorts treated between January and December 2024 (*n* = 38) and January to May 2025 (*n* = 23). Complete Δ-β2M measurements (T2 and T3) were available in 54 patients from the derivation cohort, 17 patients from the 2024 validation cohort, and 17 patients from the 2025 validation cohort. Eligible patients were ≥ 18 years with confirmed B-cell malignancies treated with commercially available CD19- and BCMA-directed CAR T-cell products. All patients received standard lymphodepletion with fludarabine and cyclophosphamide prior to infusion. CRS and ICANS were graded according to American Society for Transplantation and Cellular Therapy (ASTCT) consensus criteria [7]. ICU admission was based on institutional standardized clinical practice and generally occurred in patients developing CRS or ICANS grade ≥ 3 according to ASTCT consensus criteria. In exceptional cases, ICU admission could also occur at physician discretion in the setting of clinical deterioration before formal grade ≥ 3 toxicity was met.

Dynamic biomarkers were calculated using predefined time points: admission for CAR T-cell therapy (T1), day of infusion (T2), and day + 3 (± 1 day) after infusion (T3). Δ-values were defined as absolute changes between time points, with particular focus on Δ-β2-microglobulin (Δ-β2M) between T2 and T3. The exploratory analysis included 14 candidate laboratory parameters (CRP, albumin, LDH, creatinine, EASIX, log2EASIX, m-EASIX, platelets, fibrinogen, β2-microglobulin, ferritin, NT-proBNP, troponin and sIL2), which were assessed as baseline and/or dynamic variables depending on availability at the predefined study time points. In addition, several clinical variables extracted from the electronic health records were included in the exploratory assessment, including coronary heart disease, hypertension, diabetes mellitus, prior neurological disease, autoimmune disease, nicotine use, arrhythmia, and previous autologous stem cell transplantation. Incomplete Δ-β2M data were mainly attributable to unavailable paired β2-microglobulin measurements at the predefined study time points, reflecting the retrospective nature of routine clinical practice rather than systematic exclusion of patients. The primary endpoint was ICU admission as a surrogate for severe CAR T-related toxicity, while secondary endpoints included severe CRS and ICANS (grade ≥ 3). Associations between candidate predictors and ICU admission were assessed using logistic regression models, and predictive performance was evaluated using receiver operating characteristic (ROC) analysis. Model calibration was assessed using the Hosmer - Lemeshow goodness-of-fit test and Brier score, with cross-validated calibration plots. Clinical utility was evaluated using decision curve analysis to estimate net benefit across a range of clinically relevant threshold probabilities.

Among 97 patients in the derivation cohort, 35 patients (36%) required ICU admission. Median age was 64.2 years (interquartile range [IQR] 58.1–69.7), the median number of prior therapy lines was 3 (IQR, 2–4) and the median ICU stay among admitted patients was 3 days (IQR, 1–4) (see Table 1). High-grade CRS occurred in 17 patients (17.5%) and severe ICANS in 20 patients (20.6%). Several baseline clinical and laboratory parameters were associated with ICU admission in univariable analyses, including lower albumin levels (odds ratio [OR] 0.17, 95% confidence interval [CI] 0.07–0.46, *p* = 0.0004), higher baseline C-reactive protein (OR 1.02, 95% CI 1.00–1.04, *p* = 0.032), and elevated NT-proBNP (OR 1.001, 95% CI 1.000–1.002, *p* = 0.028). Baseline creatinine was not elevated (0.85 mg/dL (IQR, 0.73–1.08)). Although lower albumin was significantly associated with ICU admission in the derivation cohort, neither albumin nor the other baseline variables demonstrated predictive performance in the validation cohorts comparable to that observed for Δ-β2M.

**Table 1:** Baseline patient and treatment characteristics of the derivation and validation cohorts

Variable	Derivation cohort	2024	2025
Number of patients	97	38	23
Age in years, median (IQR)	64.2 (58.1–69.7)	62.9 (55.5–70.8)	63.7 (54.6–69.1)
Sex, male/female	52 (54%) / 45 (46%)	22 (58%) / 16 (42%)	19 (83%) / 4 (17%)
Number of prior therapies, median (IQR)	3 (2–4)	2 (1–3)	2 (1–3)
Diagnosis			
DLBCL	56 (58%)	17 (45%)	10 (43%)
MM	20 (21%)	9 (24%)	9 (21%)
MCL	12 (12%)	4 (11%)	
FL	4 (4%)	4 (11%)	
others	5 (5%)	4 (11%)	2 (9%)
CAR T product			
Yescarta	53 (55%)	24 (63%)	10 (43%)
Abecma	23 (24%)	9 (24%)	0
Carvykti	0	3 (8%)	9 (39%)
Tecartus	13 (13%)	1 (3%)	2 (9%)
Kymriah	8 (8%)	1 (3%)	0
Lisocel	0	0	1 (4%)
Outcomes			
CRS ≥ 3	17 (18%)	7 (18%)	2 (9%)
ICANS ≥ 3	20 (21%)	7 (18%)	6 (26%)
ICU admission	35 (36%)	14 (37%)	8 (35%)

*CRS,* cytokine release syndrome; *ICANS,* immune effector cell-associated neurotoxicity syndrome

Dynamic laboratory parameters provided superior predictive performance. Most notably, rising β2-microglobulin levels between infusion and day + 3 emerged as the strongest dynamic predictor. In ROC analyses, Δ-β2M demonstrated discrimination for ICU admission with an area under the curve (AUC) of 0.67 in the derivation cohort (*n* = 54). Importantly, this association remained consistent in temporally independent validation cohorts, with AUC values of 0.70 (*n* = 17) in 2024 and 0.82 (*n* = 17) in 2025. In contrast, Δ-β2M showed minimal discrimination for predicting ICANS, suggesting specificity for identifying patients at risk of requiring ICU-level supportive care (Fig. 1).

**Fig. 1:**
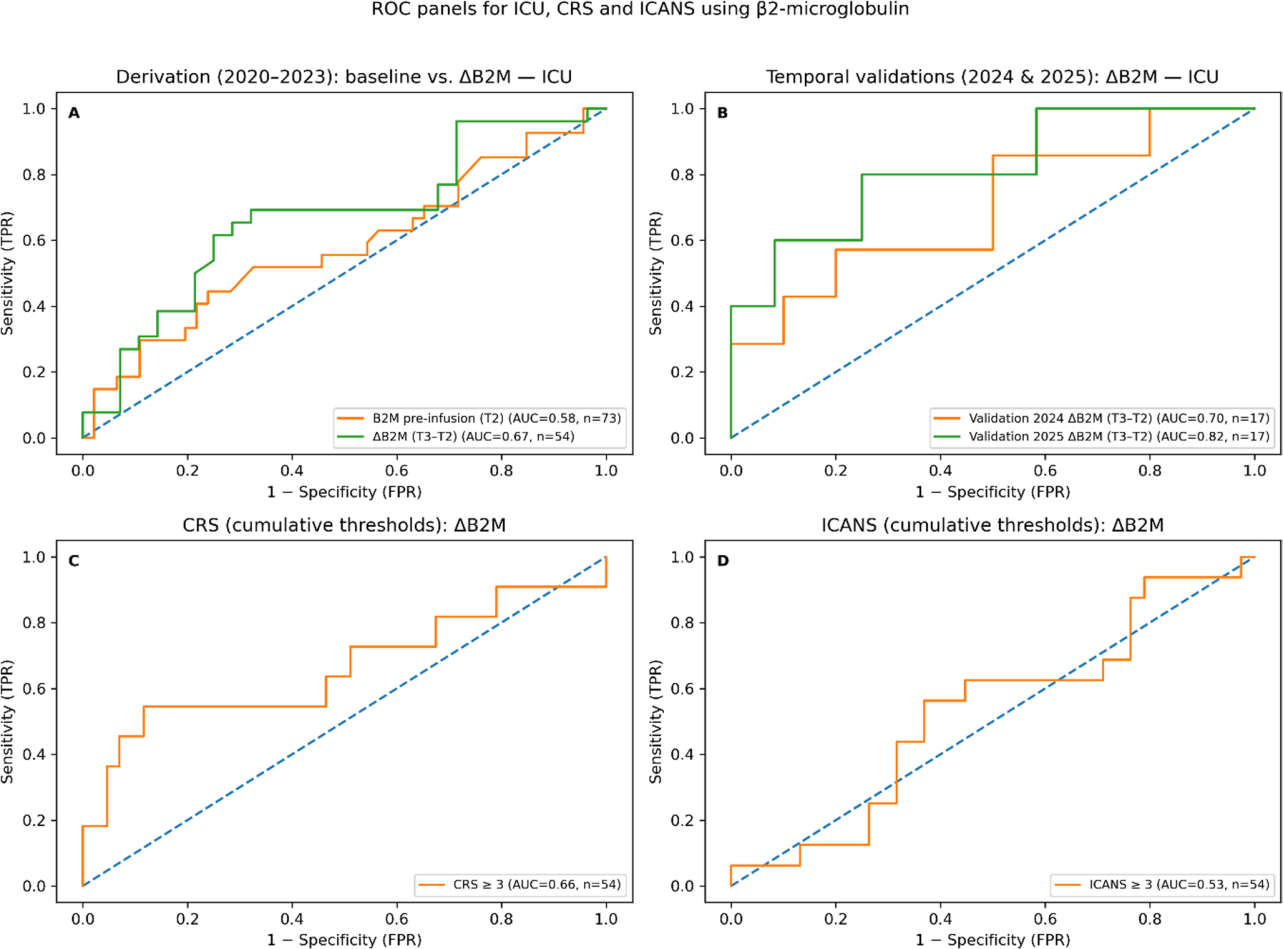
Receiver operating characteristic (ROC) curves for Δ-β2M (T3–T2) predicting ICU admission after CAR T-cell therapy. **A** Derivation cohort (2020–2023, *n* = 54). Δ-β2M demonstrated an area under the curve (AUC) of 0.67 (95% CI 0.53–0.81). **B** Temporal validation cohort 2024 (*n* = 17) with AUC 0.70 (95% CI 0.45–0.95). **C** Temporal validation cohort 2025 (*n* = 17) with AUC 0.82 (95% CI 0.62–1.00). The diagonal dashed line represents the line of no discrimination (AUC 0.5). In contrast, Δ-β2M showed no meaningful discrimination for ICANS (AUC 0.53)

To assess the clinical applicability of Δ-β2M, we performed calibration and decision curve analyses in the derivation cohort (*n* = 54 with complete Δ-β2M). The model demonstrated good calibration (Hosmer-Lemeshow *p* = 0.073; Brier score 0.232), indicating excellent agreement between predicted and observed ICU admission probabilities. Cross-validated calibration confirmed model stability (Brier score 0.236). Decision curve analysis revealed that using Δ-β2M to guide ICU admission decisions provides a positive net benefit across a range of clinically relevant threshold probabilities (0.2–0.7), with maximum net benefit achieved near the Youden index threshold of 0.49 (Fig. 2). These findings support the clinical utility of Δ-β2M for early risk stratification after CAR T-cell therapy.

**Fig. 2:**
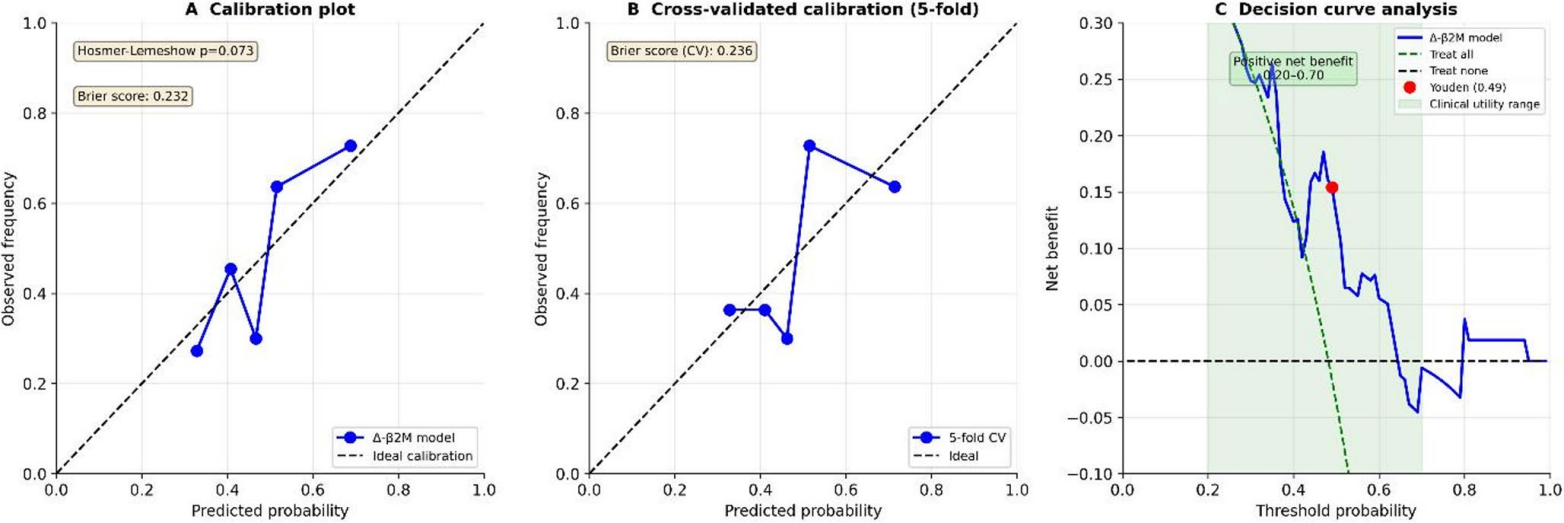
Calibration and decision curve analysis for the Δ-β2M model in the derivation cohort (*n* = 54). **A** Calibration plot showing excellent agreement between predicted and observed ICU admission probabilities. The blue line represents the Δ-β2M model, with points indicating observed frequencies across five quantile bins. The dashed diagonal line indicates ideal calibration. The model demonstrated good calibration (Hosmer-Lemeshow goodness-of-fit test *p* = 0.073; Brier score 0.232). **B** Cross-validated calibration plot (5-fold) confirming model stability (Brier score 0.236). **C** Decision curve analysis demonstrating the net benefit of using Δ-β2M to guide ICU admission decisions across a range of threshold probabilities. The Δ-β2M model (blue line) shows positive net benefit compared to “treat all” (green dashed line) and “treat none” (black dashed line) strategies for threshold probabilities between 0.20 and 0.70. The red dot indicates the Youden index threshold (0.49). The green shaded area highlights the range of clinical utility

The biological plausibility of Δ-β2M as a toxicity biomarker is supported by β2M’s role as a surrogate of cellular turnover, immune activation, and tumor burden in aggressive B-cell malignancies [8]. Baseline β2M levels did not influence ICU risk, suggesting that dynamic rather than static measurements more accurately capture early inflammatory trajectories after CAR-T infusion. In contrast, Δ-β2M demonstrated minimal utility for predicting ICANS, consistent with evidence linking neurotoxicity to endothelial dysfunction and blood–brain barrier injury [9]. Albumin and NT-proBNP provided exploratory signals but lacked consistent validation across cohorts.

This study has several limitations. The retrospective, single-center design limits generalizability and introduces potential selection bias. Another limitation is the incomplete availability of paired Δ-β2M measurements at the predefined study time points, reflecting the retrospective nature of routine clinical practice and potentially limiting generalizability. Additionally, although temporal validation strengthens reproducibility, external multicenter validation will be required to define clinically actionable thresholds and determine whether Δ-β2M can guide early intervention strategies or ICU resource allocation. The calibration and decision curve analyses were performed on the derivation cohort only (*n* = 54) and require validation in larger, independent cohorts. Furthermore, patients with advanced organ dysfunction are often excluded from CAR T-cell therapy, which may limit applicability to broader populations.

In summary, Δ-β2-microglobulin between infusion and day + 3 represents a promising and easily measurable dynamic biomarker associated with ICU admission following CAR T-cell therapy. The biomarker demonstrates good calibration and positive net benefit in decision curve analysis, supporting its potential clinical utility. While these findings are promising, external multicenter validation will be necessary before Δ-β2M can be considered for routine clinical implementation. These results support further investigation of early biomarker kinetics as components of future biology-informed and dynamically adaptive toxicity risk stratification models in CAR T-cell recipients.

## Data Availability

The data that support the findings of this study are available from the corresponding author upon reasonable request. The data are not publicly available due to privacy restrictions.

## Declarations

### Use of AI-assisted language tools

The authors used ChatGPT (OpenAI) for language editing and stylistic improvement of the manuscript text. All scientific content, data interpretation, and final manuscript revisions were performed and approved solely by the authors.
